# Unveiling the antimicrobial, biofilm inhibition, and photoprotective potential of Bupleurum falcatum L. for dermatological applications

**DOI:** 10.17179/excli2025-8344

**Published:** 2025-07-11

**Authors:** Mirjana Milinkovic Sreckovic, Jovana Petrovic, Marija Ivanov, Uros Gasic, Milena Milivojevic, Danijela Stanisavljevic Ninkovic, Dejan Stojkovic

**Affiliations:** 1 Clinic of Dermatovenereology, University Clinical Center of Serbia, Belgrade, Pasterova 2, 11000, Belgrade, Serbia; 2 Faculty of Medicine, University of Belgrade, dr Subotica starijeg 8, 11000 Belgrade, Serbia; 3 Institute for Biological Research "Sinisa Stankovic", National Institute of the Republic of Serbia, University of Belgrade, Bulevar despota Stefana 142, 11108 Belgrade, Serbia; 4 Institute of Molecular Genetics and Genetic Engineering, University of Belgrade, Vojvode Stepe 444a, PO Box 23, 11010 Belgrade, Serbia

**Keywords:** Bupleurum falcatum, skin infections, biofilm, cytotoxicity, photoprotective effect

## Abstract

*Bupleurum falcatum* L. is known for its therapeutic properties, especially in treating fever, inflammation, and infectious diseases. However, its potential for dermatological applications remains mainly unexplored. Thus, the present study explores antimicrobial potential of *B. falcatum* against biofilm-associated infections, antibiotic-resistant strains, and UV-induced skin damage. This aligns with the growing interest in natural products as sources of bioactive compounds with skin-protecting features. Herein, we employed maceration (M) and ultrasound-assisted extraction (USA) at 50 Hz and 100 Hz (USA 50 and USA 100) to obtain extracts from aerial parts of the plant. Chemical profiling was performed using UHPLC. Antimicrobial activity, biofilm inhibition, EPS and eDNA production were assessed using microdilution test, crystal violet, Congo red, and eDNA assays, respectively. Cytotoxicity and photoprotective effects were evaluated on human keratinocytes using the MTT assay. Chemical analysis identified 64 compounds, including benzoic and cinnamic acid derivatives, flavonoid glycosides, and saikosaponins. Extracts showed strong antimicrobial activity against *Pseudomonas aeruginosa* and methicillin-resistant *Staphylococcus aureus* (MIC as low as 0.5 mg/mL). The M extract displayed moderate biofilm inhibition and reduced eDNA production. Cytotoxicity assays confirmed safety on keratinocytes, while M and USA 100 extracts demonstrated photoprotective effects. *B. falcatum* extracts showed promising potential in addressing biofilm-associated infections, antibiotic resistance, and UV-induced skin damage.

See also the graphical abstract(Fig. 1).

## Abbreviations

AMR: antimicrobial resistance

DMEM: Dulbecco's Modified Eagle Medium

DMSO: dimethyl sulfoxide

DNA: deoxyribonucleic acid

eDNA: extracellular DNA

EPS: exopolysaccharide

FBS: fetal bovine serum

FWHM: full-width at half maximum definition

HaCaT: spontaneously immortalized human keratinocyte cell line

HESI-II: heated electrospray ionization

M: macerated sample of *B. falcatum*

MALDI-TOF: matrix-assisted laser desorption ionization time-of-flight

MBC: minimal bactericidal concentration

MIC: minimal inhibitory concentration 

MRSA: methicillin-resistant *Staphylococcus aureus*

MTT: 3-(4, 5-dimethylthiazolyl-2)-2, 5-diphenyltetrazolium bromide

PBS: phosphate buffered saline

TSB: tryptic soy broth

UHPLC: Ultra High Performance Liquid Chromatography.

USA 100: Ultrasound assisted sample of *B. falcatum *100 Hz

USA 50: Ultrasound assisted sample of *B. falcatum *50 Hz

UV: ultraviolet

UVB: ultraviolet B

YPD: yeast peptone dextrose

## Introduction

Antimicrobial resistance (AMR) poses a significant global health challenge, particularly in the treatment of skin infections caused by resistant pathogens. These infections are often difficult to manage due to various adaptive mechanisms pathogens use to evade drugs, such as genetic mutations and plasmid-mediated gene transfer, which allow them to neutralize antimicrobials, modify drug targets, or bypass their effects (Dadgostar, 2019[8]). Resistant strains, such as methicillin-resistant *Staphylococcus aureus* (MRSA), complicate the treatment of conditions ranging from superficial infections to severe cases like necrotizing fasciitis, particularly in immunocompromised patients (Findley and Grice, 2014[10]). This not only prolongs disease duration, but also increases healthcare costs and the likelihood of complications (Gilham et al., 2024[12]). Furthermore, the mis- and overuse of antibiotics further exacerbate this issue, necessitating immediate action to explore alternative solutions (Ahmed et al., 2024[1]). Natural-derived substances represent a promising approach for addressing AMR in skin infections. With diverse bioactive compounds, plants represent untapped source of bioactives with potential in developing safe and effective antimicrobial agents able to target resistant strains while supporting wound healing (Schwarz et al., 2006[30]). These novel alternatives could mitigate the limitations we face when using traditional antimicrobials, providing innovative therapeutic options to combat resistant skin infections (Marston et al., 2016[21]). 

In addition to antimicrobial resistance contributing to infections, increased UV radiation has increased the need for effective skin protection. This has led to growing interest in natural compounds for sunscreens, as their UV-absorbing, antioxidant, anti-inflammatory, and immunomodulatory properties offer a safer alternative to conventional photoprotective agents. Conventional sunscreens are widely used for UV protection, but concerns remain about their effectiveness in preventing skin aging and damage caused by prolonged UV exposure (Oliveira et al., 2024[26]). Due to their antioxidant properties, plant extracts emerged as potential efficient photoprotective agents. 

The rising prevalence of skin disorders caused by microbial infections and UV-induced skin damage highlights the urgent need for innovative therapeutic approaches that simultaneously address both issues, offering a novel solution combining antimicrobial efficacy with UV protection. In combination with novel delivery systems and patient-specific approaches, it seems that it could be possible to create tailored, effective skin treatments that align with the growing demand for holistic healthcare solutions (Ding et al., 2022[9]; Castaldo et al., 2024[5]). 

*B. falcatum* L. (sickled rabbit's ear) is a perennial herb from the Apiaceae family, native to Europe and Western Asia, known for its use in traditional medicine to treat infections, fever, and liver diseases (Wang et al., 2011[38]). *Bupleurum* species, documented in *Shen Nong's Herbal Classic* over 2000 years ago, have been widely used in the traditional medicine. *Bupleuri Radix* is a key ingredient in many formulations, demonstrating excellent therapeutic effects for common ailments. Longdan Xiegan Decoction, a popular remedy for chronic hepatitis in Taiwan, reflects its widespread use. The *Pharmacopoeia of the People's Republic of China *(2010) lists about 60 formulas containing *Bupleuri Radix*, which is effective in treating fever, flu, inflammation, infectious diseases, and other conditions (Yao et al., 2013[42]). Roots appear to be the most commonly used part of the plant for their health-promoting properties, largely due to the presence of bioactive saikosaponins known for their anti-inflammatory, anti-allergic, and analgesic effects (Lu et al., 2012[20]; Wang et al., 2018[39]). In Iranian folk medicine, essential oil of the root is used as a natural analgesic to treat headache and chest pain (Zargari, 1995[43]; Avicenna, 1999[3]). *B. falcatum* has also been found to possess antidepressant properties, mediated through the serotonergic and noradrenergic systems (Lee et al., 2012[19]). Strong antimicrobial potential of essential oils of *Radix Bupleuri* has been described by Ashour et al. (2009[2]) and Mohammadi et al. (2014[22]), though extracts have never been explored for this type of activity. As for the UV protective activity, *B. falcatum* extracts have never been explored, which opens new avenues for developing innovative, natural, safe, and effective dermatological therapies.

Although the roots of *B. falcatum* have been the primary focus of research due to their well-documented bioactive compounds, other parts of the plant have remained largely unexplored. Considering the growing burden of skin-related conditions and the demand for safer, nature-based therapies, we investigated *B. falcatum* as a potential natural solution. Our study aimed not only to assess its effectiveness in addressing skin disorders but also to evaluate how different extraction methods influence the chemical profile, yield, and associated bioactivities. This approach was intended to identify the most promising extract for the development of natural therapeutics targeting various dermatological conditions. Thus, herein we used maceration and ultrasound-assisted extraction at 50 Hz and 100 Hz, to identify the most efficient technique for isolating bioactive compounds from aerial parts of the plant. Chemical characterization of the extracts was performed using UHPLC to identify the most abundant compounds. Antimicrobial activity was evaluated using microdilution method, while antibiofilm effect was assessed using the crystal violet assay. Furthermore, we evaluated underlying mechanisms of antibiofilm action by studying their effects on exopolysaccharide production (Congo red assay) and extracellular DNA (eDNA) production in biofilms to navigate us to promising targets for further research. Finally, cytotoxicity of the extracts was evaluated on HaCaT skin cell line using the MTT method to confirm their safety for topical application. In addition, photoprotective effects of *B. falcatum* extracts were evaluated on HaCaT cell line.

## Material and Methods

### Plant material

Aerial parts of the *B. falcatum* plant in the flowering phase were collected in August 2023 in Vlasina, Republic of Serbia and identified by botanist Dr Dejan Stojković. Plant material was cleaned and naturally dried in the shade. The material was deposited at the Herbarium of the Institute for Biological Research “Siniša Stanković”, and the voucher number assigned was BF2023/V8. Dried material was cut into smaller pieces and grounded in a blender. The obtained powder was further used for the preparation of 70 % ethanol extracts.

### Cell culture

HaCaT immortalized human keratinocyte cell line (ATCC, Manassas, VA) was cultured in high-glucose Dulbecco's Modified Eagle Medium (DMEM) supplemented with 10 % fetal bovine serum (FBS), 2 mM l-glutamine, and 1 % antibiotic-antimycotic (all from Invitrogen™, NY, USA) under 10 % CO₂ at 37 °C.

### Preparation of plant extracts

Plant extracts of *B. falcatum* were prepared using maceration and ultrasound-assisted extraction methods to compare their yield, chemical profile and bioactivities. For maceration, plant powder (3 g) was mixed with 100 mL of 70 % ethanol and stirred at room temperature (25 °C) for 24 h (Welp Scientifica, Usmate, Italy). For ultrasound-assisted extraction, two samples (3 g each) were prepared using the same solvent after which they were processed using an ultrasonic bath (Bandelin Sonorex Digiplus, Berlin, Germany) at 25 °C for 30 minutes, one at 50 Hz and the other at 100 Hz. After extraction, all samples were concentrated to dryness using a rotary vacuum evaporator (Büchi Labortechnik AG R-21, Switzerland). Finally, we obtained three extracts: macerated (further in the text labeled as M), ultrasound-assisted 50 Hz (further in the text labeled as USA 50) and ultrasound-assisted 100 Hz (further in the text labeled as USA 100).

### Chemical analysis of B. falcatum extracts 

Metabolic profiling of *B. falcatum* extracts was conducted using an LC-HRMS/MS system (Thermo Scientific™ Vanquish™ Core HPLC coupled with the Orbitrap Exploris 120 mass spectrometer, San Jose, CA, USA) as described previously by Zengin et al. (2020[44]). The liquid chromatography system utilized a Hypersil GOLD™ C18 analytical column (50 × 2.1 mm, 1.9 μm particle size), maintained at 40 °C. The injection volume was 5 μL, and the flow rate was set at a constant 300 μL/min. Compound separation was achieved using a gradient elution with ultrapure water containing 0.1 % formic acid (solvent A) and MS-grade acetonitrile with 0.1 % formic acid (solvent B): 5 % B for 1 minute, 5-95 % B from 1 to 10 minutes, 95 % B from 10 to 12 minutes, and returning to 5 % B until 15 minutes. The Orbitrap Exploris 120 was equipped with a heated electrospray ionization (HESI-II) source operating in both positive and negative ionization modes. Operating conditions included a spray voltage of ± 4500 V, capillary temperature of 325 °C, and an RF tube lens voltage of 50 %. Nitrogen was used as the sheath gas (30 AU), auxiliary gas (10 AU), and sweep gas (1 AU). Full-scan MS spectra were collected from 100 to 1500 m/z with a resolution of 60,000 FWHM. Data-dependent MS^2^ experiments were performed at a resolution of 15,000 FWHM, with a normalized collision energy of 35 %, an isolation width of 1.5 m/z, and a dynamic exclusion time of 10 seconds after two occurrences. The intensity threshold was set at 1 × 10⁵.

### Antimicrobial activity

#### Isolation and identification of pathogenic bacteria and fungi from skin 

Swab samples containing microorganisms from the skin were collected from patients with skin infection at Clinic of Dermatovenereology, University Clinical Center of Serbia. Eleven samples were collected from human volunteers, with informed consent obtained for their use in the present study. Microorganisms were identified using the Vitek MS system (bioMérieux SA, Marcy-l'Étoile, Lyon, France), which applies matrix-assisted laser desorption ionization time-of-flight (MALDI-TOF) technology for automated identification of microorganisms through mass spectrometry. Samples were prepared according to guidelines provided by manufacturer for the analysis.

#### Antibacterial and anticandidal activity

Antibacterial properties were evaluated using microdilution method described previously by Soković et al. (2009[34]). Assay was performed on both resistant and non-resistant clinical isolates of bacteria. Among Gram-positive, following strains were used: Methicillin resistant *Staphylococcus aureus* IBRS 011, *S. aureus* IBRS1 (skin isolate), *S. aureus* IBRS2 (skin isolate), *S. aureus* IBRS3 (skin isolate), *S. aureus* IBRS4 (skin isolate). As for the Gram-negative bacteria, we used: *Escherichia coli* IBRS E003, *Pseudomonas aeruginosa* IBRS1 (skin isolate), *P. aeruginosa* IBRS2 (skin isolate), *P. aeruginosa* IBRS3 (skin isolate), *P. aeruginosa* IBRS4 (skin isolate), *P. aeruginosa* IBRS P001 (skin isolate from animals), *Citrobacter koseri *(skin isolate), and *Proteus mirabilis* (skin isolate). Among yeasts, *Candida albicans* IBRS1 (skin isolate) was used. All the microorganisms are deposited in the Culture collection of the Mycological Laboratory, Department of Plant Physiology, Institute for Biological Research “Siniša Stanković” - National Institute of Republic of Serbia, University of Belgrade. Methicillin resistant *S. aureus* IBRS 011, *E.coli* IBRS, *P. aeruginosa* IBRS P001 were isolated as described in Kartsev et al. (2018[16]), whereas *S. aureus* IBRS1, *S. aureus* IBRS3, *S. aureus* IBRS3, *S. aureus* IBRS4,* P. aeruginosa* IBRS1, *P. aeruginosa* IBRS2, *P. aeruginosa* IBRS3, *P. aeruginosa* IBRS4, *C. koseri*, *P. mirabilis, *and *C. albicans* IBRS1 were isolated and identified as described in section: Isolation and identification of pathogenic bacteria and fungi from skin. Cultures of microorganisms were grown overnight at 37 °C in TSB/YPD and adjusted to a density of 1.0 × 10⁵ CFU/mL using sterile saline. Test samples, dissolved in 30 % ethanol, were added to TSB medium (100 μL) along with bacterial inocula (1.0 × 10⁴ CFU per well). After overnight incubation at 37 °C, *p*-iodonitrotetrazolium chloride solution (0.2 mg/mL, 40 μL) was added to each well. Subsequently, plates were incubated for an additional 60 minutes at 37 °C to allow color to develop. The minimal inhibitory concentration (MIC) was defined as the lowest sample concentration that reduced in color intensity (light red compared to the deep red in untreated controls) or complete absence of color. Minimal bactericidal concentration (MBC) was determined after serial sub-cultivation, with 2 μL from each well being transferred to fresh broth and incubated for 24 hours at 37 °C. The MBC was identified as the lowest concentration that eradicated bacterial growth, indicating a 99.5 % reduction of the initial inoculum. Streptomycin (1 mg/mL, for bacteria) and itraconazole (1 mg/mL, for yeast) were used as positive controls.

#### Antibiofilm activity

Antibiofilm activity assay was conducted as described previously by Živković et al. (2015[45]) with some modification. Subinhibitory concentrations of extracts M and USA 50 were tested using 96-well flat-bottom microtiter plates. *P. aeruginosa* IBRS001 was adjusted to 10^6^ CFU/mL and incubated with serially diluted extracts in TSB enriched with 2 % glucose at 37 ˚C for 24 h. After incubation, the wells were washed twice with sterile PBS (pH 7.4), air-dried, and the residual biofilm was fixed with methanol (100 µL) for 10 minutes. The biofilm was then stained with crystal violet (0.1 %, 100 µL) for 10 minutes, after which excess stain was removed by washing with distilled water. Finally, the remaining dye bound to the biofilm was solubilized by adding 95 % ethanol (200 µL). After 10 minutes the absorbance was measured using Multiskan™ FC Microplate Photometer, Thermo Scientific™. The levels of biofilm production were calculated as follows:

*% production = 100 x A* *_620_* *sample/A* *_620_* *control*

A_620_ sample = absorbance of sample treated with *B. falcatum* extract

A_620_ control = absorbance of untreated biofilm

#### Congo red test

Assessment of *B. falcatum* extracts impact on the production of exopolysaccharides (EPS) as essential components of biofilm was determined using the Congo red dye binding assay, according to the protocol previously described by Ivanov et al. (2022[13]). Bacterial biofilms of *P. aeruginosa* IBRS P001 were formed in the presence of *B. falcatum* (MIC, 0.5 MIC and 0.25 MIC) at 37 °C, for 24 h. Planktonic cells were removed and adherent cells were washed with PBS. The wells were stained with Congo red dye (1 %, 100 μL, SigmaAldrich, Germany). After incubation, wells were aspirated and the bounded dye was dissolved with DMSO (100 μL). Absorbance was measured at 490 nm and results calculated by following equation:

*% production = 100 x A* *_490_* *sample/A* *_490_* *control*

A_490_ sample = absorbance of sample treated with *B. falcatum* extract

A_490_ control = absorbance of untreated biofilm

#### Extracellular DNA (eDNA) assay

eDNA assay was used to quantify extracellular DNA as an essential component of biofilm as described by Selvaraj et al. (2021[31]) with some modifications. *P. aeruginosa* IBRSP001 was grown in 96-well plates (Sarstedt, Germany) containing TSB medium supplemented with 2 % glucose and *B. falcatum* extract (MIC, 0.5 MIC, and 0.25 MIC) at 37 °C, for 24 h. After overnight growth, the planktonic cells were removed, and the wells were thoroughly rinsed with PBS. TE buffer (Tris-EDTA buffer solution) was added to the samples and mixed vigorously by pipetting and scraped. The samples were transferred to 1.5 mL tubes and centrifuged at 8000 rpm for 10 min to settle the biofilm. After discarding the supernatant, the pellet was re-suspended into a TE buffer and vortexed. The quantification of eDNA (ng/μL) in supernatant was measured on NanoPhotometer^®^ N60 (Implen, Munich, Germany) after centrifugation at 12,000 rpm for 15 min. The levels of eDNA in biofilm matrix were evaluated according to the following equation:

*% production = 100 x C* *_sample_* */ C* *_control_*

C_sample_ = concentration of DNA within treated biofilm sample

C_control_ = concentration of DNA within untreated biofilms

#### Cytotoxicity towards HaCaT skin cell line

Cytotoxicity was evaluated using MTT assay. For this assay, *B. falcatum* extracts were diluted in PBS. Cells (1.7 x 10⁴ per well) were seeded into 96-well plates with adhesive bottoms; the next day, medium was removed, and cells were treated with various concentrations of the test samples. Cell viability was assessed 24 h after the treatments using 3-(4,5-dimethylthiazol-2yl)-2,5-diphenyltetrazolium bromide (MTT) assay (Merck KGaA, Gernsheim, Germany). MTT solution was added to cell cultures at a final concentration of 0.5 mg/mL and cells were incubated with solution for 1 h at 37 °C. Following the incubation, MTT solution was removed and cells were lysed with DMSO (Serva Electrphoresis GmbH, Heidelberg, Germany). Reduction of a yellow tetrazolium salt to purple formazan crystals by metabolically active cells was observed using a microplate reader (BioTek Epoh, Agilent, USA) at a 550 nm wavelength. Cell's viability was calculated in relation to viability of treated cells without treatment. Experiment was performed at least in triplicate.

#### UVB irradiation of cells

For irradiations of HaCaT cells, we used custom made solar simulator whose ssUV spectrum comprised 5.5 % UVB and 94.5 % UVA, closely matching the spectrum of natural sunlight. For UVB irradiation, 1.7 x 10⁴ HaCaT cells were seeded into 96-well plates per well and next day were treated with 100 µM *B. falcatum* extracts. 24 h after the treatment, cells were washed with PBS and a thin layer of PBS was left in plates, exposed to 50 and 150 mJ/ cm^2^ of UVB and incubated in a fresh complete medium for the next 24 h. Upon incubation, cell viability was assessed using MTT assay as described above. Experiment was performed at least in triplicate.

### Statistical analysis

Experiments were repeated three times, and results are presented as means ± standard errors. The data was statistically analyzed using GraphPad Prism 9.0.0. The differences between control and experimental groups were compared using Multiple *t*-test. Values *p* (*p<0.05, **p<0.01, ***p<0.001, ****p<0.0001) were considered to be statistically significant.

## Results and Discussion

Yields of *B. falcatum* extracts, namely M, USA 50 and USA 100 are presented in Table 1(Tab. 1). The yield of the *B. falcatum* extract obtained through maceration (M) was 21.45 % (w/w), while ultrasound-assisted extraction at 50 Hz (USA 50) and 100 Hz (USA 100) resulted in yields of 4.40 % (w/w) and 12.90 % (w/w), respectively. These results indicate that maceration, a traditional extraction method, was the most effective in achieving the highest yield. However, even though maceration of the plant tissue resulted in the highest yield of dry matter, better bioactive properties of the ultrasound-assisted extracts (presented further in the text) suggest that the latter extraction method provides faster and more efficient cell wall disruption. This, in turn, facilitates the release of bioactive compounds, resulting in greater overall bioactive potential, which will be elaborated later on. 

**Figure 2:**
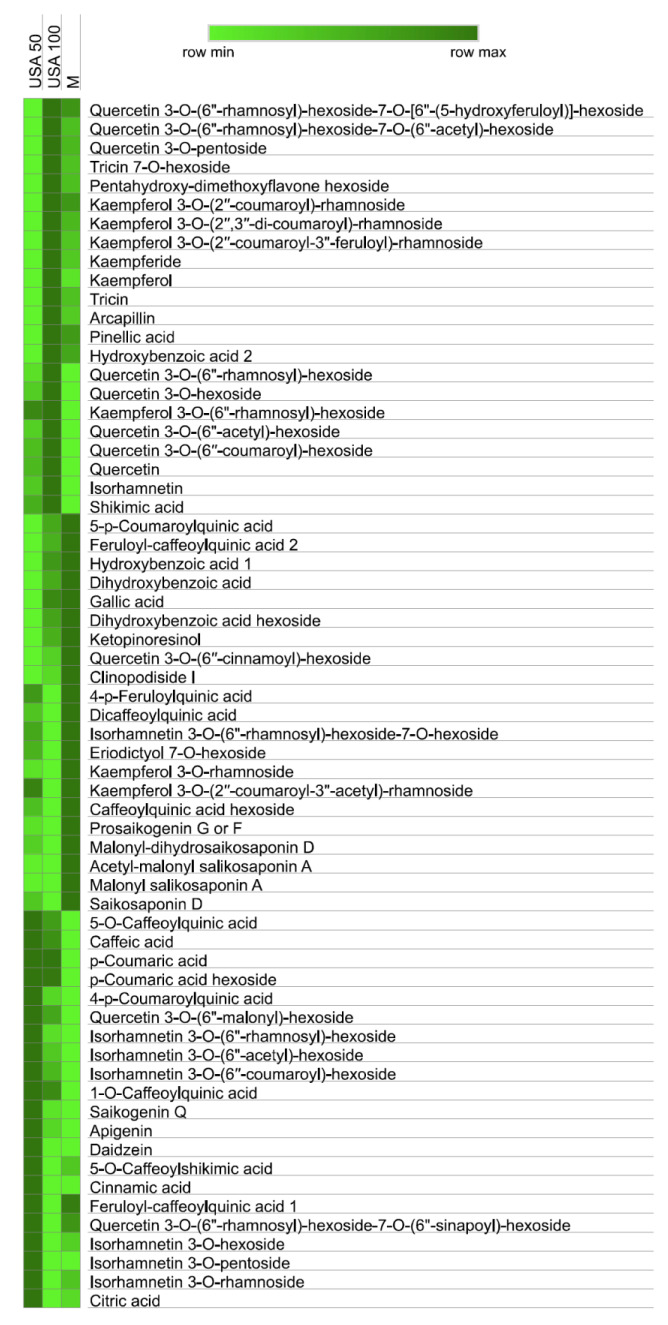
Heat map representing compound distribution in M, USA 50 and USA 100 samples based on peak compound abundance presented in Supplementary material.

Chemical profiling showed a total of 64 compounds identified in *B. falcatum* extracts. All identified compounds with retention times and main characteristics of mass spectrometry i.e. exact mass and MS^2^ fragmentation are presented in Supplementary Table S1. Their identification was based on comparison with corresponding standards where available, and with published data on metabolites previously identified in the *B. falcatum* plant. Detected compounds were divided into the following groups: phenolic acids, including benzoic and cinnamic acid derivatives (19 compounds), flavonoid glycosides and aglycones (34 compounds), saponins (7 compounds) and other phenolic compounds (4 compounds). The abundance of compounds identified in all three samples is vividly illustrated in the heat map, clearly highlighting both the differences and similarities in their chemical composition (Figure 2(Fig. 2)). In the sample USA 50, we characterized following compounds: kaempferol 3-*O*-(6"-rhamnosyl)-hexoside, 4-*p*-feruloylquinic acid, kaempferol 3-*O*-(2′′-coumaroyl-3"-acetyl)-rhamnoside, 5-*O*-caffeoylquinic acid, caffeic acid, *p*-coumaric acid, *p*-coumaric acid hexoside, 4-*p*-coumaroylquinic acid, quercetin 3-*O*-(6"-rhamnosyl)-hexoside, isorhamnetin derivatives (3 in total, see Figure 2(Fig. 2)), 1-*O*-caffeoylquinic acid, saikogenin Q, apigenin, daidzein, 5-*O*-caffeoylshikimic acid, cinnamic acid, feruloyl-caffeoylquinic acid 1, quercetin 3-*O*-(6"-rhamnosyl)-hexoside-7-*O*-(6"-sinapoyl)-hexoside, isorhamnetin 3-*O*-hexoside, isorhamnetin 3-*O*-pentoside, isorhamnetin 3-*O*-rhamnoside and citric acid. Contrary to this, sample USA 100 exhibited a high abundance of different compounds, albeit within the same group: quercetin 3-*O*-(6"-rhamnosyl)-hexoside-7-*O*-[6"-(5-hydroxyferuloyl)]-hexoside, quercetin 3-*O*-(6"-rhamnosyl)-hexoside-7-*O*-(6"-acetyl)-hexoside, quercetin 3-*O*-pentoside, tricin, tricin 7-*O*-hexoside, pentahydroxy-dimethoxyflavone hexoside, kaempferol derivatives (in total 3 compounds, see Figure 2(Fig. 2)), kaempferide, kaempferol, arcapillin, pinellic acid, hydroxybenzoic acid 2, quercetin and quercetin hexoside derivatives (5 in total, see Figure 2(Fig. 2)), isorhamnetin, shikimic acid, *p*-coumaric acid and *p*-coumaric acid hexoside. Similar trend can be observed in the third macerated sample. Thus, in sample M, following compounds had the highest abundance: 5-*p*-coumaroylquinic acid, feruloyl-caffeoylquinic acid 1, hydroxybenzoic acid 1, dihydroxybenzoic acid, gallic acid, dihydroxybenzoic acid hexoside, ketopinoresinol, quercetin 3-*O*-(6′′-cinnamoyl)-hexoside, clinopodiside I, 4-*p*-feruloylquinic acid, dicaffeoylquinic acid, eriodictyol 7-*O*-hexoside, kaempferol 3-*O*-rhamnoside, kaempferol 3-*O*-(2′′-coumaroyl-3"-acetyl)-rhamnoside, caffeoylquinic acid hexoside, prosaikogenin G or F, malonyl salikosaponin A, acetyl-malonyl salikosaponin A, malonyl-dihydrosaikosaponin D, and saikosaponin D. The presence of saponins in *B. falcatum* is anticipated, given it has been previously described by Park et al. (2002[28]). This indicates the importance these compounds have in the plant's bioactive composition. Our obtained results are overall in accordance with those published by Tung et al. (2015[36]), where saikosaponins A and D, flavonoids, monoterpene glycosides and quinic acid derivatives, were detected in ethanolic extract of *B. falcatum* aerial parts. However, saponins have been identified in the ethanolic extracts of *B. falcatum* roots as well (Park et al., 2000[27]).

Antimicrobial activity of *B. falcatum* extracts was evaluated against different bacterial isolates, including those resistant to antimicrobial therapeutics (Table 2(Tab. 2)). Obtained results suggest that all three analyzed extracts possess quite significant activity towards all the tested microorganisms. The most significant result was achieved with extracts M and USA 50 on resistant strain of *P. aeruginosa*. These extracts demonstrated strong antibacterial activity with MIC and MBC values of 0.5 mg/mL and 1 mg/mL, respectively. Similar inhibitory and bactericidal effects were observed against methicillin-resistant *S*.* aureus* and resistant *E. coli* strains as well, with MIC and MBC values of 2 mg/mL and 4 mg/mL. Our results are in accordance with previously published data regarding antimicrobial potential of *Bupleurum *spp. Thus, Laouer et al. (2009[18]) demonstrated that essential oil from *B. montanum* and *B. plantagineum* inhibit growth of *S. aureus*. Furthermore, *B. lancifolium* extracts demonstrated notable antibacterial and antifungal properties, as was investigated by Shafaghat (2011[32]). Regarding antifungal potential, essential oil of *B. falcatum* showed moderate antifungal potential towards *Aspergillus flavus*, *Alternaria alternata, *and* Fusarium oxysporum* (Nesic et al., 2023[23]).

Over the past few decades, plant extracts and essential oils have generally demonstrated diverse bioactivities, particularly in the eradication of pathogenic microorganisms, making them valuable candidates for the development of novel antimicrobials. Antimicrobial properties of plants have mainly been attributed to phenolic acids, terpenes, and organic acids, which often act synergistically to enhance their efficacy (Vaou et al., 2021[37]). Considering that several phenolic compounds identified in our extracts are known for their antimicrobial properties, it is likely that they contribute to the observed antimicrobial activity. These compounds are considered to act by disrupting bacterial cell membranes, increasing permeability, and impairing essential metabolic processes. In particular, their small size enhances the ability to penetrate bacterial cells and exert antimicrobial effects (Cushnie and Lamb, 2005[7]). In addition to directly inhibiting the growth of pathogenic microorganisms, our extracts, which are rich in phenolic compounds, could also serve as potential adjuvants in conventional antimicrobial therapy. Many phenolic compounds have shown the ability to enhance the effectiveness of traditional antibiotics through synergistic effects.

This could increase their medicinal value, especially in situations where conventional antibiotics are no longer effective, making them an important consideration in future drug development (Cha et al., 2016[6]; Nguyen and Bhattacharya, 2022[24]). However, further research is essential to comprehend and confirm their additional therapeutic value.

**Figure 3:**
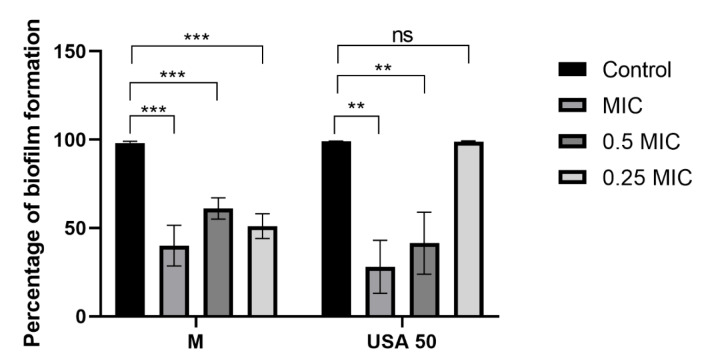
Percentage of biofilm biomass production determined in CV assay, results are presented as mean value ± standard deviation (SD) of three replicates ** p ≤ 0.01, *** p ≤ 0.001, ns - non significant p > 0.05.

Beyond their direct antimicrobial effects, plant extracts also exhibit strong antibiofilm activity, providing a promising strategy for combating infections that persist in biofilm form. For the evaluation of antibiofilm capacity we have selected 2 extracts, M and USA 50, based on their lower values of MICs towards *P. aeruginosa* IBRSP001 (Table 2(Tab. 2)). In our study, *B. falcatum* extracts M and USA 50 exhibited significant antibiofilm activity, except for the USA 50 extract at 0.25 MIC, which did not cause significant changes in biofilm levels (Figure 3(Fig. 3)). On the other hand, sub-inhibitory concentration (0.5 MIC) for both M and USA 50 significantly reduced biofilm biomass levels to 61.08 % and 41.41 %, respectively. Interestingly, the activity was not strictly dose-dependent, with varying effects at different concentrations. Our study is the first one to explore antibiofilm activity of *B. falcatum* extracts, with flavonoids and saponins likely contributing to their effects, as they are known to inhibit bacterial adhesion and reduce extracellular polysaccharide synthesis (Gao et al., 2021[11]). The ability to disrupt biofilm formation is crucial in the context of rising antimicrobial resistance, as biofilms protect bacteria from harsh environments and enhance their survival (Slobodníková et al., 2016[33]; Tatli Cankaya and Somuncuoglu, 2021[35]). By targeting biofilm development, plant-derived extracts offer new potential strategies to combat chronic and persistent bacterial infections, which are often resistant to conventional treatments (Watnick and Kolter, 2000[40]). In more details, since biofilms provide a protection that increases bacterial survival in harsh environment, by targeting key steps in biofilm development we could increase possibility for complete bacterial eradication (Ivanov et al., 2022[14]). 

**Figure 4:**
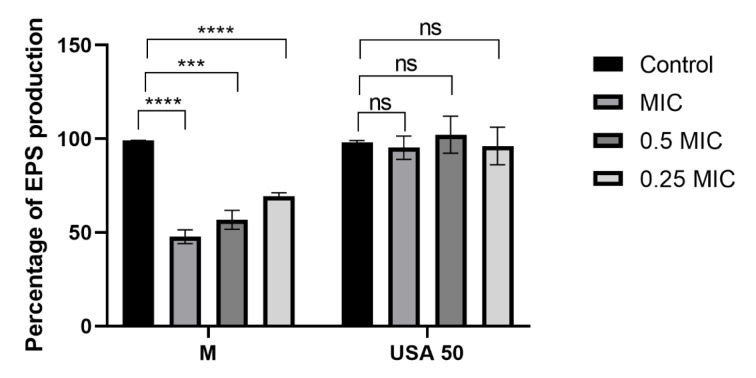
Percentage of biofilm exopolysaccharide (EPS) production results are presented as mean value ± standard deviation (SD) of three replicates **** p ≤ 0.0001, *** p ≤ 0.001, ns - non significant p > 0.05.

To gain deeper insights into the effect of *B. falcatum* extracts on the biofilm-forming capacity of treated bacteria, and to assess the potential of plant-derived compounds as biofilm inhibitors, we employed the Congo red dye binding test. The obtained results shown in Figure 4(Fig. 4) indicate that incubation with all the examined concentrations of extract M induced significant changes in levels of EPS within biofilms. Even at the lowest tested concentration of M (0.25 MIC) levels of EPS reached 69.35 %. On the other hand, extract USA 50 did not significantly affect EPS levels, suggesting that a different antibiofilm mechanism underlies its activity. Supporting evidence from Ivanov et al. (2022[15]) and Carević et al. (2024[4]) highlights the significant inhibitory effects of flavonoids, such as rutin, diosmin, neohesperidin, and myricetin on exopolysaccharide production, suggesting their potential role in destabilizating biofilm. This implies that different flavonoids, such as quercetin derivatives present in extract M (Figure 2(Fig. 2)), might be responsible for the observed inhibitory effect. Further research is required to clarify the mechanisms and optimize the use of biofilm inhibition as an alternate approach in treating persistent and chronic bacterial infections.

**Figure 5:**
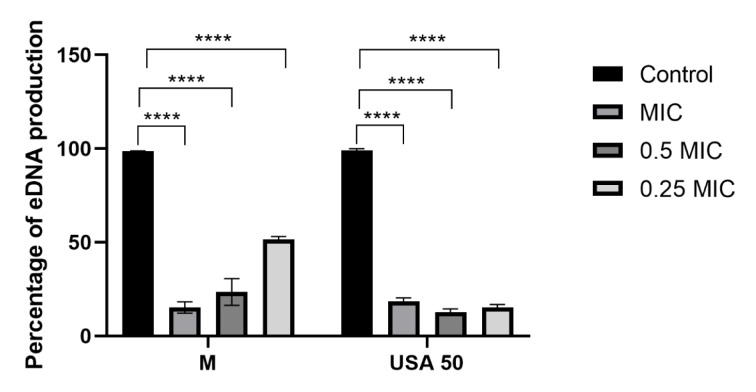
Figure 5: Percentage of biofilm eDNA production, results are presented as mean value ± standard deviation (SD) of three replicates **** p ≤ 0.0001, *** p ≤ 0.001.

The other possible approach in disrupting biofilm formation is to affect eDNA production. Our results regarding the effect of *B. falcatum* extracts on eDNA production in *P. aeruginosa* biofilm are presented in Figure 5(Fig. 5). Obtained data revealed that extract M in all the tested concentrations had strong inhibitory effect on eDNA production. It can be noticed that applied extract was least efficient using the lowest concentration (0.25 MIC). On the other hand, effect on eDNA production using extract USA 50 was more consistent with potent inhibition of eDNA production, and the lowest levels of eDNA produced at 0.5 MIC (12.81 %). Since eDNA is a key component in the *P. aeruginosa* biofilm matrix, playing a crucial role in biofilm stability, bacterial adhesion, and antibiotic resistance (Sarkar, 2020[29]), our results suggest that natural extracts from *B. falcatum* can effectively inhibit this critical factor, highlighting their potential in disrupting microbial biofilms. The results presented herein are consistent with previous studies, which have shown that enzymatic degradation of eDNA can prevent biofilm formation and reduce bacterial resistance (Okshevsky and Meyer, 2015[25]). However, further research is needed in order to better understand and validate the mechanism underlying this process. 

**Figure 6:**
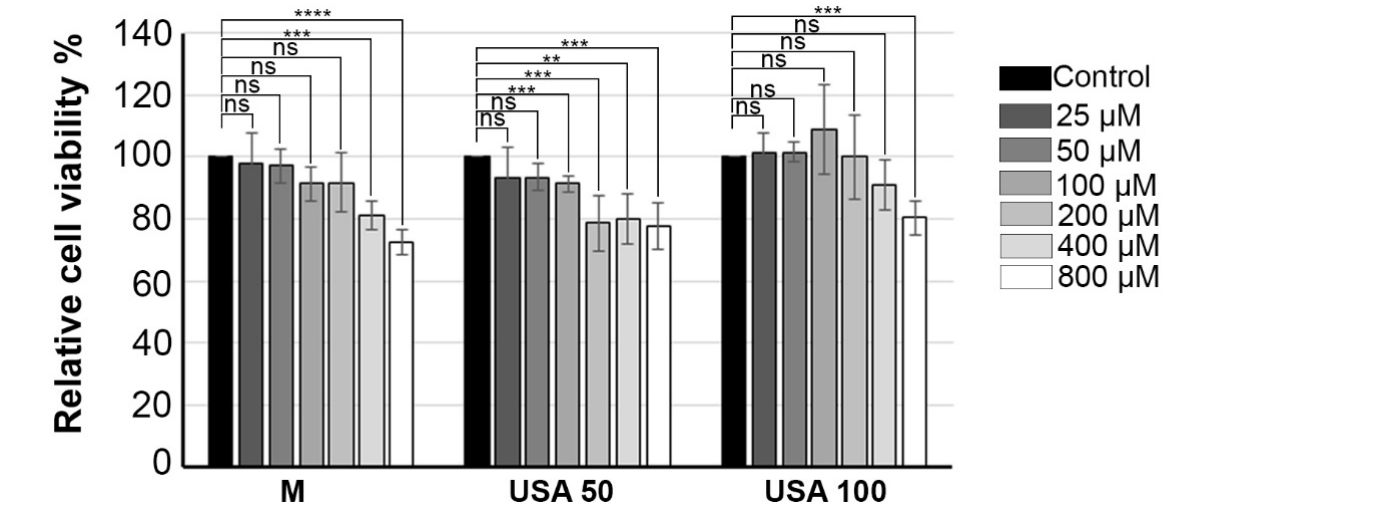
Effect of increased concentration (0, 25, 50, 100, 200, 400 and 800 µM) of *B. falcatum* extracts on HaCaT cell's viability. Cells without treatment was designated as Control. 24 h after treatment cell viability was determined using MTT assay. Relative cell's viability for cells treated with each *B. falcatum* extracts was calculated as a percentage of cell line viability compared to control without treatment (designated as control) that was set as 100%. Results were presented as the means ± SEM of at least three independent experiments. *p* values were calculated using Student's t test, **** *p* ≤ 0.0001, *** *p* ≤ 0.001, ** *p* ≤ 0.01.

When exploring plant-derived antimicrobial agents, assessing safety and cytotoxicity of the extracts is crucial, particularly for their topical use in skin treatments. In this study, immortalized human keratinocyte HaCaT cell line was used to analyze *in vitro* cytotoxic activity of *B. falcatum* extracts. This cell line was treated with various concentration of *B. falcatum* extracts M, USA 50 and USA 100, and 24 h upon treatment, cell viability was determined using the MTT assay. As shown in Figure 6(Fig. 6), all the tested *B. falcatum* extracts showed slight statistically significant cytotoxicity at the highest applied concentration (800 µM) and the percent of viable cells remained above 72 % under all conditions. In particular, treatment with extract M significantly decreased cell viability by 20 % at concentration of 400 µM and by 28 % at concentration of 800 µM compared to control. Furthermore, treatment of HaCaT cells with extract USA 50 at concentrations exceeding 200 µM resulted in a significant 20 % decrease in cell viability compared to the control. On the contrary, a significant reduction (20 %) in cell viability was observed with extract USA 100 only at a concentration of 800 µM, compared to the control cells. Thus, it can be concluded that *B. falcatum* extracts do not exert a cytotoxic effect on HaCaT cells (Figure 6(Fig. 6)); therefore extracts are safe for topical application on human skin, which may be of practical importance in treating skin infections given their demonstrated high efficiency towards skin pathogenic microorganisms. 

**Figure 7:**
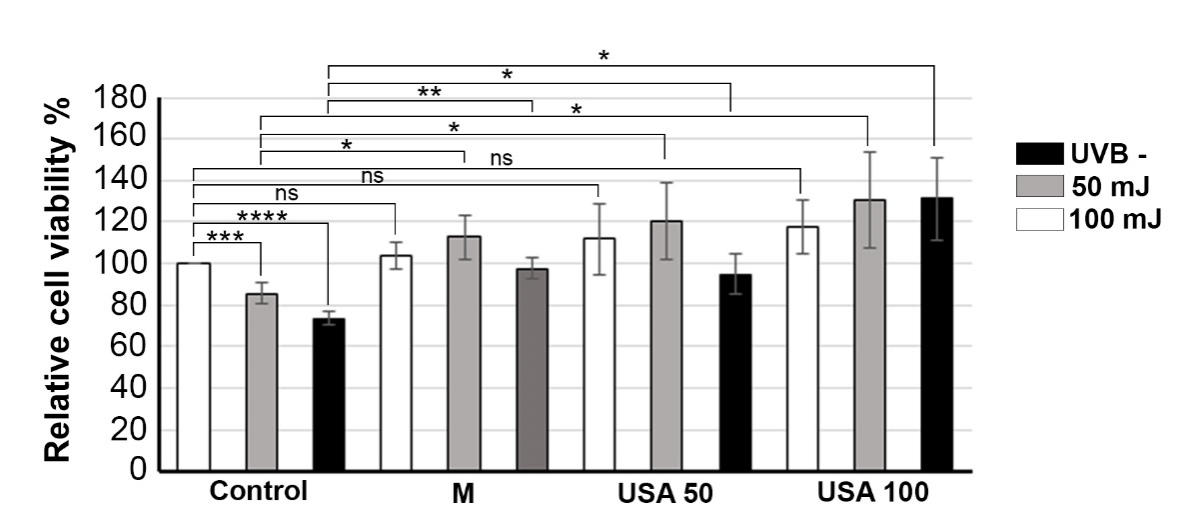
Photoprotective effect of *B. falcatum* extracts on cell viability in UVB-exposed human keratinocytes. Results were presented as the means ± SEM of at least three independent experiments. *p* values were calculated using Student's t test, **** *p* ≤ 0.0001, *** *p* ≤ 0.001, ** *p* ≤ 0.01, *p ≤ 0.05.

To evaluate the UVB properties of *B. falcatum* extracts on skin, human keratinocytes were treated with extracts (M, USA 50 and USA 100) 24 h prior to UVB irradiation. Cell viability was then assessed the following day to determine the extracts' protective effects. As it was shown in Figure 7(Fig. 7), pre-treatment with 100 µM of all tested extracts, particularly M, showed statistically significant photoprotective effect on HaCaT cells against both 50 mJ/cm^2^ and 150 mJ/cm^2^ UVB. Particularly, pre-treatment with extracts M, USA 50 and USA 100 led to significant increased viability by 27 %, 34 % and 45 % respectively, compared to cells irradiated with 50 mJ/cm^2^ UVB. Further, compared to cells irradiated with 150 mJ/cm2 UVB, pre-treatment with extracts M, USA 50 and USA 100 led to significant increased viability by 25 %, 22 % and 58 %, respectively. Our data suggest that *B. falcatum* extracts effectively prevented photodamage by protecting skin keratinocytes against UVB-induced cytotoxicity, suggesting its potential use in skin protection against UVB-induced damage.

Overall, our results showed that tested samples of *B. falcatum* had promising inhibitory effects towards multidrug-resistant *P. aeruginosa*. As discussed, this activity can be attributed to the chemical profile of extracts, which are rich in phenolic compounds. These, on the other hand may disrupt cell membranes or interfere with the essential enzymatic pathways, ultimately leading to cell death, as concluded by Xiao and Gao (2024[41]). Additionally, phenolics are well-known for their ability to inactivate bacteria (Kauffmann and Castro, 2023[17]). This is further supported by our findings, where the extracts inhibited biofilm formation through two distinct mechanistic pathways. Specifically, extracts demonstrated ability to disrupt production and structural integrity of key biofilm components, such as eDNA and polysaccharides. By targeting these two essential biofilm components, *B. falcatum* may destabilize biofilms and offer a promising strategy in managing *P. aeruginosa* infections, particularly those involving biofilms which contribute to chronic course of disease, such as in non-healing wounds, chronic venous leg ulcers, or burn-related infections. Through disruption of biofilm, extract could also allow better diffusion of active compounds and/or co-administered antibiotics. In addition to this, extracts maintained cell viability, and demonstrated significant photoprotective effects against UVB-induced damage, indicating their potential for safe and effective dermatological applications, particularly with respect to skin infections and skin damage caused by UV radiation.

See also the supplementary data.

## Conclusion

In conclusion, *B. falcatum* demonstrated significant potential as a natural antibacterial and antibiofilm agent, particularly against *P. aeruginosa*, MRSA, *E. coli* and *C. albicans* strains. The chemical analysis revealed extracts are rich source of bioactive compounds, which contribute to very good antimicrobial and antibiofilm activities. Extracts effectively inhibited biofilm formation, reduced exopolysaccharide and eDNA production, and showed promising antibacterial effects at low concentrations. Futhermore, these extracts showed no toxicity towards human keratinocytes, suggesting their safety for topical application. Pre-treatment with all tested extracts show significant photoprotective effect on HaCaT cells against UVB irradiation. Based on our showing antimicrobial and anti-biofilm properties of *B. falcatum* against skin pathogenic microorganisms, particularly *P. aeruginosa*, we suggest that it holds strong potential for dermatological applications in addressing difficult-to-treat skin infections caused by this pathogen. Ultimately, these findings highlight the potential of *B. falcatum* in combating bacterial infections, particularly drug-resistant strains, and emphasize the need for further studies to support its safe and effective application in dermatological therapies.

## Notes

Mirjana Milinkovic Sreckovic and Jovana Petrovic contributed equally to this work.

## Declaration

### Acknowledgment

We acknowledge the use of ChatGPT [https://chat.openai.com/] in helping us to review English writing at the final stage of preparing the manuscript.

### Funding

This research is funded by the Serbian Ministry of Science, Technological Development and Innovation [Contract No. 451-03-136/2025-03/200007, 451-03-66/2024-03/200042 and Grant No. 451-03-66/2024-03/200110]. Authors would like to appreciate the contribution of MSc Nikoleta Premović, who performed some of presented experiments for her master thesis.

### Data availability statement

The data that support the findings of this study are available on request from the corresponding author [J.P.].

### Declaration of interest

Authors disclose no conflict of interest.

### Author contributions

Conceptualization [JP and MMS], data curation [MI and UG], formal analysis [MMS, MI, DSN and UG], investigation [JP, MMS and MM], methodology [MM, MI and DS], supervision [MMS and DS], validation [JP], writing - original draft [JP, MMS and MI], writing - review and editing [JP and MM].

## Supplementary Material

Supplementary information

Supplementary data

## Figures and Tables

**Table 1:**
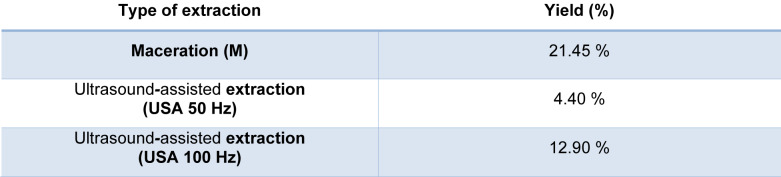
Yield of *B. falcatum* extracts (%)

**Table 2:**
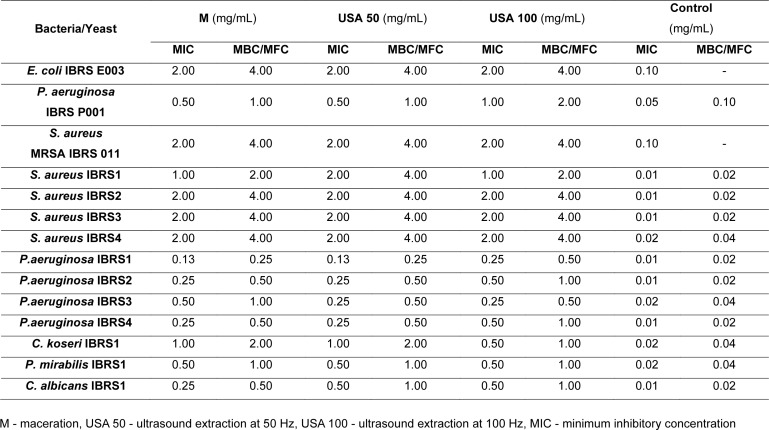
Antimicrobial activity of B. falcatum extracts (M, USA 50 and USA 100; mg/ml)

**Figure 1:**
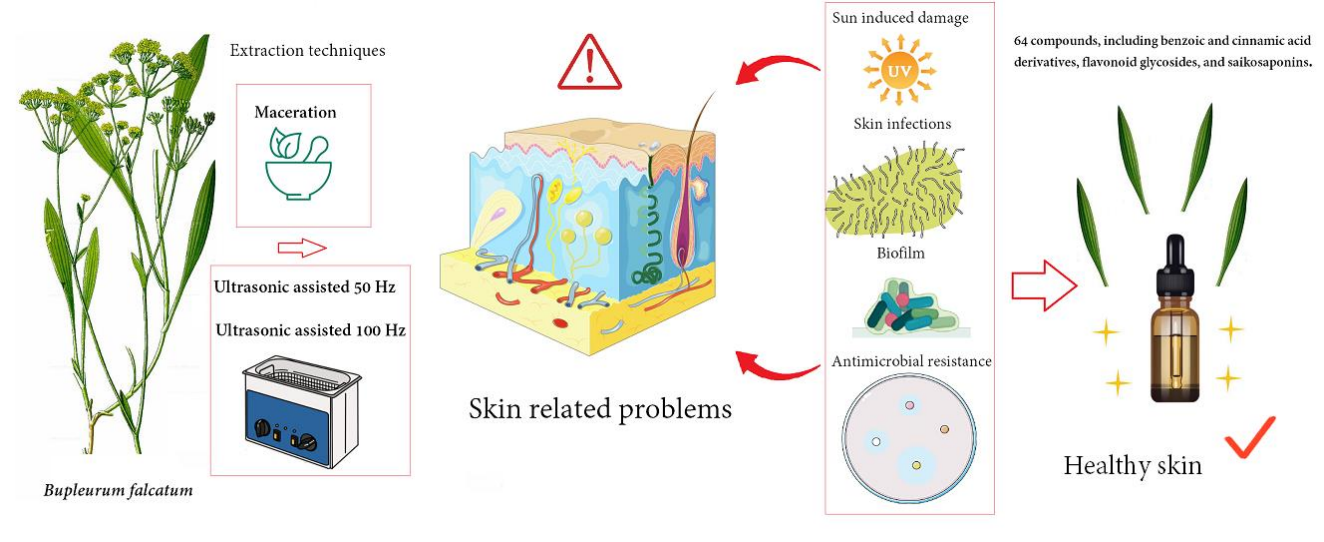
Graphical abstract
